# Development and testing of a new electronic foot health promotion programme on nurses’ foot self-care

**DOI:** 10.1186/s12912-020-00423-z

**Published:** 2020-04-19

**Authors:** Minna Stolt, Jouko Katajisto, Johanna Peltonen, Riitta Suhonen, Helena Leino-Kilpi

**Affiliations:** 1 Department of Nursing Science, University of Turku, and Turku University Hospital, Turku, Finland; 2 Department of Mathematics and Statistics, University of Turku, Turku, Finland; 3 Department of Nursing Science, University of Turku, Turku, Finland; 4 Department of Nursing Science, University of Turku, and Turku University Hospital, City of Turku, Welfare Division, Turku, Finland

**Keywords:** Foot health, Foot self-care, Intervention, Nurses, Work ability

## Abstract

**Background:**

Nurses form the largest professional group in health care, and they spend most of their working day on their feet. From the perspective of work well-being, healthy feet are important to tolerate the physical demands of nursing work. However, little is known about how nurses’ foot self-care practices can be promoted with computerised interventions. The aim of this study was two-fold: to explore the preliminary effects of the electronic Foot Health Promotion Programme (FHPP) on foot self-care in nurses and to examine the usability of the programme.

**Methods:**

A single group pretest-posttest design was used. The FHPP was targeted at nurses working in the operating theatre. The FHPP lasted for 4 weeks and focused on improving nurses’ knowledge and awareness of foot self-care through self-directed learning tasks. The primary outcome was knowledge of foot self-care. The secondary outcomes were foot health and work ability. Thirty-seven participants completed the study. The outcomes were assessed at baseline (April–June 2017) and 4 weeks (August–September 2017) after the intervention ended. The data were analysed statistically.

**Results:**

Participants’ knowledge of foot self-care and foot health improved; however, the changes were not statistically significant. The FHPP was considered to be usable and has potential as a tool to increase knowledge of foot self-care among nurses.

**Conclusions:**

The FHPP developed in this study is a newly developed potential tool to increase nurses’ knowledge of foot self-care. Application of the FHPP as part of occupational health care may enhance nursing personnel’s foot self-care and lower extremity health.

**Trial registration:**

ClinicalTrials.gov NCT03116451, 17.4.2017.

## Background

Foot self-care (caring for one’s own feet) is important for maintaining and improving foot health and, in turn, general health. Foot health is one aspect of physical health. Physical health and its promotion are emphasised in international strategies and guidelines [1, 2]. However, in these guidelines, foot self-care and foot health are rarely mentioned as central enabling factors for physical health. Supporting foot self-care is commonly believed to be a prerequisite for maintaining and improving foot health; therefore, there is a need to include foot self-care in these guidelines.

Foot health is important throughout life. In general, foot health is appreciated, and attitudes towards the care of foot problems are often positive [3]. However, foot problems are common in working life among nurses [4] and can decrease quality of life, particularly among females [5]. Nurses form the largest professional group in health care. Nursing is physically demanding, including situations in which nurses’ feet are exposed to stress for long periods in their daily work environment. However, studies focusing on supporting and improving nurses’ foot health are limited [4].

Foot self-care is performed by individuals. It requires knowledge of how to look after oneself properly and practical skills in caring for one’s own feet. Foot self-care involves regular hygiene, toenail and skin care, foot exercises and the use of appropriate footwear and socks [6]. Despite its rather straightforward content, foot self-care is often overlooked. Reasons for this may include lack of knowledge [7]; physical restrictions, such as arthritis [8] or obesity [9]; lack of motivation [10]; or the perception that caring for the feet is not important [11]. On the other hand, social support, foot-related education and communication between patients and providers have been reported to enable foot self-care [12].

Interventions focusing on foot self-care are scarce [13], and previous studies have focused on improving foot self-care in patients with diabetes [14, 15]. The interventions studied were all educational, and they included key areas of diabetic foot self-care. Educational interventions were provided in groups [16–18] and in face-to-face settings [16, 17]. They were reinforced by written material [14], lectures and meetings with health-care professionals [14, 19] or periodic support and foot checks [20]. The individual sessions varied from 20 min [21] to 90 min [16], and the full interventions lasted from 20 min [21] to 4 weeks [14]. Only a limited number of interventions were conducted in an electronic format, which included text messages [22] and audio-visual lectures [16]. These interventions resulted in improved knowledge of foot self-care, which confirms that foot self-care can be enhanced through an educational approach. However, there is limited evidence of how foot self-care can be improved through electronic programmes in occupational health settings, specifically among nurses.

Previous studies of foot self-care have focused on patients with long-term health problems [19]. However, little is known about foot self-care in professionals whose feet are exposed to stress for long periods in their daily work environment. One such group is nurses. In health care, nurses form the largest professional group [23], and they have higher rates of musculoskeletal disorders (MSDs) than people in all other occupations [24, 25]. Nurses spend most of their working day on their feet, often standing and walking on concrete or other hard surfaces. In clinical nursing practice, nurses need to stand and walk for long periods; it is estimated that nurses walk 4 to 5 miles in a 12-h shift [26]. All these factors increase the number of musculoskeletal disorders in nurses [27]. Nurses working in the operating theatre have to stand for long durations, often in poorly fitting footwear, which poses a threat to foot health. Footwear for standing environments should be selected individually, and the corresponding nurses’ foot health status and functionality of the footwear should match with the environment [28].

Previous prevalence studies of MSDs have found that the proportion of knee, ankle and foot disorders in nurses is high. The prevalence of MSDs ranges from 22.8 to 68.7% in knees and from 9.3 to 53.4% in ankles and feet. In addition, leg fatigue and foot discomfort are prevalent in nurses [29, 30]. Health problems caused by maintaining an upright posture for long durations include plantar fasciitis, muscle fatigue, varicose veins [31] and oedema [32]. The intensity of foot pain in nurses is high [33], and this intercorrelates with increased age and higher body mass index [34]. Moreover, the use of footwear that does not support foot health has a negative impact on general wellbeing at work [35]. Nurses tend to wear shoes that have lost their structural integrity, which increases foot pain and other foot problems [34]. All these problems in the lower extremities could be prevented and controlled by appropriate foot self-care.

Some interventions to promote general health and work ability have been tested [36, 37]. Structured workshops and periodical forums for sharing best practices are feasible and potentially useful for reducing MSDs and risk factors for work-related injuries [36]. More specifically, ergonomic interventions [37, 38], workplace exercises [39] and procedures for handling and lifting patients [40, 41] are also effective in reducing rates of MSDs. However, these studies did not focus on health in the lower extremities or on foot self-care. Therefore, interventions focusing on improving nurses’ foot self-care are needed.

The aim of this study was two-fold: to develop and explore the preliminary effects of the electronic Foot Health Promotion Programme (FHPP) on foot self-care in nurses and to examine the usability of the programme. The research questions were as follows: 1) What effect does the FHPP have on knowledge of foot self-care, foot health and work ability? 2) What is the usability of the FHPP among nurses? The hypothesis was that the FHPP is effective in increasing knowledge of foot self-care.

## Methods/Design

A single group pretest-posttest design was used. The trial was registered with ClinicalTrials.gov (identifier: NCT03116451). For the reporting of the results, the Transparent Reporting of Evaluations with Nonrandomized Designs (TREND) statement was followed [42].

The study was conducted in surgical units in one university hospital in Finland. All nurses working in these units were recruited to participate in the study. They received information about the study and, based on that, made the decision whether to participate or not. Nurses were eligible to participate if they 1) were a registered nurse; 2) had a permanent post in the hospital; 3) worked in the operating theatre; and 4) understood Finnish. A sample size of 60 was considered sufficient on the basis of a power analysis conducted using NQuery 4.0 software using repeated-measures analysis of variance (ANOVA) with a two-sided 5% significance level and a power of 80%. A total of 60 eligible participants were recruited for the study; however, only 56 participated in the intervention. During the study period, 19 participants who had completed the baseline measurement (measurement 0, later M0) withdrew from the research, which left 37 in the intervention group. Those who withdrew were not asked to give their reasons for doing so.

### Data collection

The data were collected between 04/2017 and 09/2017. All the participants were assessed for baseline data (measurement 0) and follow-up data (measurement 1) within this time frame. Measurement 1 was performed 4 weeks after the intervention ended. In total, four instruments were used.

The primary outcome of knowledge of foot self-care was measured using the modified Foot Self-Care Knowledge Test [43]. The test consisted of 20 items divided into five subscales: skin care (4 items), toenail care (4 items), structural deformities in the foot (4 items), disease-specific foot problems (4 items), and footwear (4 items). The response options were “true,” “false” and “do not know”. The total score ranged from 0 to 20; the higher the score, the higher the level of knowledge about foot self-care.

The secondary outcomes were foot health and work ability. Foot health was measured using the Self-reported Foot Health Assessment Instrument (S-FHAI). The S-FHAI is based on the clinical and objective Foot Health Assessment Instrument (FHAI) [44], which was modified to create a self-assessment form for this study. The S-FHAI consisted of 28 items divided into five subscales: skin health (12 items), toenail health (4 items), foot structure (5 items) and foot pain (7 items). The instrument produces a total sum variable (Foot Health Index) by totalling the items. The Foot Health Index ranges from 0 to 28; the higher the value, the healthier the feet. Work ability was measured by asking the participants to evaluate their current wellbeing at work on a scale from 0 to 10. The higher the value, the better their work ability [45].

In addition, information was collected about the participants’ age, gender, number of years and months working in health care, length of time working in their current unit and type of employment. Information was also gathered about the footwear worn at work and the importance of foot health in their current role.

The usability of the programme was evaluated by the participants 4 weeks after the intervention ended, at measurement 1 (August–September 2017), using a questionnaire developed for this study. The questionnaire consisted of 14 items. First, participants were asked to give the FHPP an overall rating (from 1 to 10, where 1 is lowest and 10 is highest). Second, participants were asked 10 questions about the content and layout of the FHPP, to which they responded using a 5-point Likert scale (1 = strongly disagree, 5 = strongly agree). Third, participants were asked three open-ended questions where they could freely express their feelings about the positive aspects of the FHPP, any concerns about the FHPP and any recommendations for future development.

### Intervention

Interviews [10], a survey [46], a literature review [4] and collaboration with professionals in occupational health care guided the design and content of the educational electronic FHPP for nurses. The material in the FHPP covered foot self-care and the promotion of foot health. The content was divided into four topics: skin and toenail care, footwear and hosiery, foot structure and pain, and foot muscle strength (Table 1). A pretest to ensure the clarity of the FHPP was conducted with purposefully selected nurses (*n* = 6) working in the surgical units. They tested the FHPP and provided critical feedback about the clarity and understandability of the instructions and content. Based on the feedback, the wording of the instructions was revised, and some minor amendments were made to the content.

**Table 1:** Content of the electronic Foot Health Promotion Programme (FHPP)

Foot Health Promotion Programme (FHPP) 4 weeks				
**Week**	**1**	**2**	**3**	**4**
**Theme**	**A: Skin and toenail care**	**B: Footwear and hosiery**	**C: Foot structure and pain**	**D: Foot muscle strength**
**Teaching method**				
**Lecture** (delivered via Adobe Presenter)	Opening lecture on the importance of foot health by a physiatrist and a podiatrist	Lecture on the importance of proper footwear and hosiery	Lecture on the importance of a healthy foot structure and pain-free feet	Lecture on the importance of muscle strength in everyday life and at work
**Self-directed learning** using material collected to learning platform	- Principles of skin care (text and video)	- Properties of optimal professional footwear (text)	- Foot functions and structure (text)	- Foot training and exercises (text and video)
	- Care relating to skin problems (text)		- Identifying problems with foot structure, and providing the related care (text)	
	- Care relating to foot oedema (text and video)	- How to measure footwear size correctly (text and video)		- Foot stretches (text and video)
			- Mechanisms of foot pain (text)	- Muscle balance (text)
	- Toenail care (text and video)	- Properties of optimal hosiery (text)	- Methods to alleviate foot pain (text and video)	
	- Identifying toenail problems (text)			
**Asking questions**	Each participant had the option to contact the researcher with questions by email via the learning platform			
**Evaluation**^**a**^	Test and feedback	Test and feedback	Test and feedback	Test and feedback

^a^Each respondent received an example of a correct answer and used that to evaluate their own answers

The intervention was delivered using the Moodle web-based learning environment. Each topic included lectures (delivered via Adobe Presenter), videos, photos and written material for self-directed learning. At the end of each theme section, the participants evaluated their learning by completing a knowledge test. The items in the knowledge test were related to the content of the topic, and the correct answers could be found in the material in Moodle. After completing the test, the participants received a list of the correct answers with feedback. The FHPP was available for 4 weeks. The participants used the FHPP independently, and they were able to move back and forth between the topics.

### Data analysis

The data were analysed statistically using SPSS 22.0 software (SPSS Inc., Chicago, USA). The outcome data were analysed using descriptive statistics to describe the samples and study variables. Inferential statistics were used to test for differences between the groups; these statistics included ANOVA (F with degrees of freedom and *p*-value, for organisation comparisons and the non-parametric Kruskal–Wallis test). For longitudinal analyses, the general linear model (GLM, repeated measures) for continuous variables, the T-test (Mann–Whitney U-test) for analysis between groups, and the Wilcoxon signed rank test were used to analyse changes from baseline within groups. Multivariable methods, such as regression analysis and GLM statistics, were used to examine the hypothesised association between the variables. Factor analyses and structural equation modelling were used to examine the validity of the results. The psychometrics of the instruments were evaluated using the Kuder-Richardson formula.

The data on usability were analysed using descriptive statistics (frequency, percentage, mean, standard deviation and range) and inductive content analysis [47]. All phrases and sentences in the transcribed material that included terms or words close to those in the research questions were tabulated with the intent to organise the data. Using the identified sentences, responses to the research questions were collected. The units of meaning were condensed into smaller units, and those condensed units of meaning were abstracted and named using codes. Codes with similar properties were grouped together and named based on their content. The analysis process was confirmed by the research team.

### Ethical considerations

The study was conducted in accordance with national legislation, general principles of research ethics [48] and national ethical standards [49]. Ethical approval was obtained from the university ethical review board (code: ETMK 14/2015, 23.2.2015), and permission to conduct the study was requested from the hospital in line with national standards and procedures. Each participant was informed in writing about the aim of the study; participants were also informed that their participation was voluntary, that their anonymity and confidentiality would be preserved in all phases of the study, and that they had the right to withdraw at any time without any negative consequences. Participants were also given the contact details of a member of the research team in case they wished to discuss the study in more detail. Written informed consent was obtained from each participant. To protect participants’ privacy and anonymity, the data were coded using individual identifiers so that individual respondents could not be identified. The research data were stored on a server at the university. Permission to shorten and modify the Foot Self-Care Knowledge Test was obtained from the original developer.

## Results

### Description of the study participants

The mean age of the participants was 43.4 years (range 24–61), and the majority were female (95%; see Table 2). Almost all participants considered foot health to be very important (78%) or important (16%) in their work. Three-quarters (73%) of the participants said their work involved a great deal of standing or walking. One-third (35%) of the participants had visited a physician due to foot problems, and one-fifth (19%) had been on sick leave because of foot problems. Most of the participants believed that foot health had a large impact on their work (60%).

**Table 2:** Participant demographics (*n* = 37)

Variable	Intervention group (M0)			
	***n***	Mean	range (SD)	f (%)^**a**^
Age (years)	*35*	43.43	24–61 (10.30)	
Practical experience in health care after graduation (years)	*37*	20.10	2–42 (10.80)	
Practical experience in the current unit (years)	*36*	11.51	1–31 (7.97)	
Gender	*36*			
Male				1 (3)
Female				35 (95)
Importance of foot health at work	*35*			
Very important				29 (78)
Important				6 (16)
Somewhat important				2 (6)
Amount of standing or walking at work	*37*			
A lot				27 (73)
Quite a lot				9 (24)
Not much or a little				1 (3)
Visited a physician due to foot problems	*36*			
Yes				13 (35)
No				23 (62)
Impact of foot health on work	*37*			
Very large				22 (60)
Large				6 (16)
Neither large nor small				4 (11)
Small				3 (8)
Very small				2 (5)
Sickness absence due to foot problems	*37*			
Yes				7 (19)
No				30 (81)

^a^the number of responses vary due to missing information

### Effect of the FHPP on knowledge of foot self-care, foot health and work ability

The primary outcome of the study was knowledge of foot self-care. Following the intervention, some improvement was achieved in the total score level for knowledge of foot self-care (Table 3). Therefore, overall, participants’ knowledge increased following the intervention. However, the difference was not statistically significant (*p* = 0.126). At the individual level, 23% (*n* = 13) of the participants improved their knowledge of foot self-care.

**Table 3:** Participants’ knowledge of foot self-care, foot health and work ability

Outcome	Baseline (M0) ***n*** = 37		Post-test (M1) ***n*** = 37		***p***-value*
	Mean	Range	Mean	Range (SD)	
*Primary outcome:*					
Knowledge of foot self-care	12.1	2–19 (3.01)	13.23	6–17 (2.65)	0.126
*Secondary outcomes:*					
Foot Health Index	16.68	10–32 (5.40)	19.16	9–36 (6.82)	0.109
Work ability	8.78	6–10 (1.16)	8.27	4–10 (1.49)	0.135

*T-test (Mann–Whitney U-test)

The secondary outcomes were foot health and work ability, and these improved slightly. The mean score on the Foot Health Index, which indicates the level of foot health, increased from 16.68 to 19.16 (Table 3); however, the difference was not statistically significant (*p* = 0.109). Some areas of foot health improved, but others worsened (Table 4). Instances of thickened toenails, colour changes in the toenails and hammer toes decreased considerably. On the other hand, instances of cold feet, low arches and oedema in the feet increased greatly. There were no significant differences in foot health at the item level. The intensity of foot pain increased in all areas of the foot; in particular, there was an increase in instances of strong pain in various areas of the foot when compared with the baseline data (Table 5). Pain in the thigh (*p* = 0.017) and pain in the heel (*p* = 0.003) increased significantly after the intervention. At the individual level, foot health improved in one-fifth of participants (*n* = 14, 25%).

**Table 4:** Participants’ self-reported foot health (by item)

Variable	Baseline (M0) ***n*** = 37		Post-test (M1) ***n*** = 37	
	f	%	f	%
**Skin healt**				
Skin breaks or macerations between toes	4	14	3	8
Dry skin	22	60	24	65
Heel fissures	9	24	10	27
Corns or calluses	14	38	19	51
Verrucae	0	0	0	0
Blisters	2	5	4	11
Oedema	14	38	14	38
Sweating feet	8	22	13	35
Burning feet	6	16	6	16
Cold feet	10	27	18	49
Leg cramps	13	35	15	41
**Toenail health**				
Ingrown toenail	2	5	5	14
Thickened toenail	8	22	6	16
Colour changes in the toenails	10	27	6	16
Fungal infection of the toenails	1	3	1	3
**Foot structure**				
Hallux valgus	10	27	10	27
Taylor’s bunion	5	14	7	19
Hammer toes	1	3	0	0
Low arches	8	22	16	43
High arches	1	3	4	11

**Table 5:** Foot pain in participants

Location of pain	Baseline (M0) ***n*** = 37										Post-test (M1) ***n*** = 37									
	No pain		Slight pain		Moderate pain		Strong pain		Worst imaginable pain		No pain		Slight pain		Moderate pain		Strong pain		Worst imaginable pain	
	f	%	f	%	f	%	f	%	f	%	f	%	f	%	f	%	f	%	f	%
Toes	39	70	4	11	3	9	5	9	1	3	24	65	5	14	2	5	5	14	1	3
Sole of the foot	18	49	6	16	9	24	4	11	0	0	15	41	11	30	5	14	4	11	1	3
Heel	26	70	5	14	4	11	1	3	1	2	19	51	6	16	6	16	6	16	0	0
Ankle	25	68	5	14	5	14	2	5	0	0	22	60	9	24	4	11	0	0	2	5
Knee	19	51	4	11	10	27	4	11	0	0	16	43	6	16	6	16	8	22	1	3
Thigh	31	84	3	8	1	3	0	0	1	3	22	60	5	14	5	14	3	9	1	3
Hip	24	65	7	19	4	11	1	3	1	3	17	46	8	22	6	16	4	11	0	0

The participants’ work ability decreased slightly following the intervention (*p* = 0.135).

### Usability of the FHPP

The participants gave the FHPP a rating of 6.9 out of 10 (range 4–9). Overall, the participants evaluated the usability of the FHPP as positive. The participants thought that the instructions related to the FHPP were clear (mean 4.2) and that the whole programme was easy to use (mean 4.1). They thought the content of the FHPP was up to date (mean 3.9) and versatile (mean 3.8). The technical aspects of the FHPP worked well (mean 3.7), and it was considered beneficial (mean 3.6). The FHPP inspired participants to care for their feet (mean 3.3), and they felt that the layout was attractive (mean 3.0). The participants thought that the FHPP met their expectations (mean 3.5) and needs (mean 3.5).

The participants considered that overall, the FHPP is a suitable method for providing education about foot self-care. They were satisfied with the information and especially valued the education on footwear and socks and on strength in the lower muscles. However, they identified some areas for development. One theme of the FHPP was foot exercises, and the education on this was provided in the form of written instructions and videos, where a podiatrist demonstrated the exercises. The participants thought that foot exercises were important, but they were not sure whether they were performing the exercises correctly. Therefore, the participants suggested that foot exercises could be either supervised (with more detailed instructions) or guidance could be provided in face-to-face groups by a podiatrist.

## Discussion

Interventions focusing on foot health, particularly in nurses, are limited. Therefore, the electronic Foot Health Promoting Programme (FHPP) developed in this study is novel and fills this gap in the research knowledge. In this study, the electronic FHPP demonstrated effectiveness at a descriptive level only. The FHPP improved knowledge of foot self-care and improved foot health, but the changes were not statistically significant.

The present findings support the results of previous studies: knowledge of foot self-care can be influenced by education [16, 50, 51]. To achieve a greater change in the scores for knowledge of foot self-care, a face-to-face lecture and leaflets could provide supporting information to improve participants’ knowledge levels. Changes in foot health may take longer to achieve. The post measurement (M1) was conducted 4 weeks after the intervention. This may be too short of a time to observe major changes in foot health. In the future, more measurement points (for example, at 12 and 24 weeks after the intervention) may reveal more variation in foot health.

The intervention was delivered in an online learning environment, where participants’ learning and information-gaining strategies are crucial. The participants of this study may have been active or inactive in relation to managing their own health. We did not ask the participants about their level of motivation; therefore, no significant changes were observed. In addition, influencing individuals’ motivation in an electronic learning environment is challenging. Therefore, the programme may benefit from elements of online or face-to-face practical demonstration [17] or telephone calls [50] to support participants in taking care of their feet.

Despite the lack of statistically significant results, in this research, a preventive foot-health intervention programme targeted at nurses was developed and tested. The results demonstrate the need to develop the content and delivery of the intervention further. With this development, the programme has the potential to prevent foot health problems among workers in occupational health care. The programme could be provided as part of routine health checks or targeted at those whose foot health needs special attention. If effective, the FHPP could promote nurses’ foot health and general wellbeing, leading to improvements in their work ability, wellbeing and effectiveness at work. At the organisation level, the programme could make various units more efficient due to workers’ improved foot health and general wellbeing. At the societal level, the programme would (at least partly) decrease sickness-related absences related to problems with the lower extremities. Protecting the occupational health of nurses is important if we are to guarantee a trained workforce in clinical practice.

### Limitations

Although the purpose of this study was to explore usability and provide preliminary evidence of potential effects, the study has some limitations that need to be discussed. First, the study design was a single group repeated-measures design. The study lacked a control group, but despite several attempts, it was not possible to recruit such a group. Without a control group, there is a chance that the results were affected by confounding factors such as lack of adherence or motivation towards foot self-care. Second, the sample size was limited. Only a small proportion of the total number of potential participants consented, despite several reminders and letters of encouragement. The reasons for this may include a lack of interest in foot health, a lack of time or a lack of motivation to participate. To confirm the effectiveness of the FHPP, a larger study that implements randomisation would be necessary. The generalisability of the results must be done with care. In terms of gender and professional background, the participants represented the averages for nurses working in specialised health care in Finland [52]. Third, the content of the FHPP focused on skin and toenail care, footwear and hosiery, foot structure and pain, and foot muscle strength. Comprehensive content would have been more informative, including for example, foot self-care to alleviate specific problems (such as corns, fissures or splay foot). However, the FHPP covered the core aspects of foot self-care and was supported by the expert panel.

The FHPP was developed systematically following evidence-based guidelines and recommendations on foot self-care. The content of the FHPP was reviewed by professionals in foot health care. The FHPP was delivered to each of the participants in a similar manner, and its content remained unchanged throughout the study. The participants were asked about the usability of the FHPP, and the majority believed that the content was useful and that the FHPP was easy to use. However, in the future, it would be beneficial to interview participants about their experiences of the FHPP to improve its technical functions and appearance. Moreover, user experiences of the content would be important to identify possible context-related development issues.

The instruments used in this study were all in Finnish and had been used in previous studies. The number of missing values was low, indicating that the instruments were easy to administer and that their content was understandable. The internal consistency of the S-FHAI (Kuder–Richardson coefficient 0.737) and the Foot Self-Care Knowledge Test (Kuder–Richardson coefficient 0.617) was acceptable.

## Conclusions

The Foot Health Promotion Programme is a promising tool to support nurses’ foot self-care. The results provided preliminary evidence that it is possible to increase nurses’ knowledge of foot self-care using an electronic web-based educational programme. However, further development of the educational programme is needed. In addition, the long-term or permanent increase in knowledge levels should be investigated using a follow-up design.

## Data Availability

The datasets used and/or analysed during the current study are available from the corresponding author on reasonable request.

## Abbreviations

FHPP: Foot Health Promoting Programme
MSD: Musculoskeletal disorder
TREND: Transparent Reporting of Evaluations with Nonrandomised Designs
